# An epigenetic master regulator teams up to become an epioncogene

**DOI:** 10.18632/oncotarget.16484

**Published:** 2017-03-22

**Authors:** Jianfei Qi, Fabian Volker Filipp

**Affiliations:** Systems Biology and Cancer Metabolism, Program for Quantitative Systems Biology, University of California Merced,Merced,CA, USA

**Keywords:** epigenomics, cancer systems biology, precision medicine, master regulator, transcription factor network

**Figure 1:**
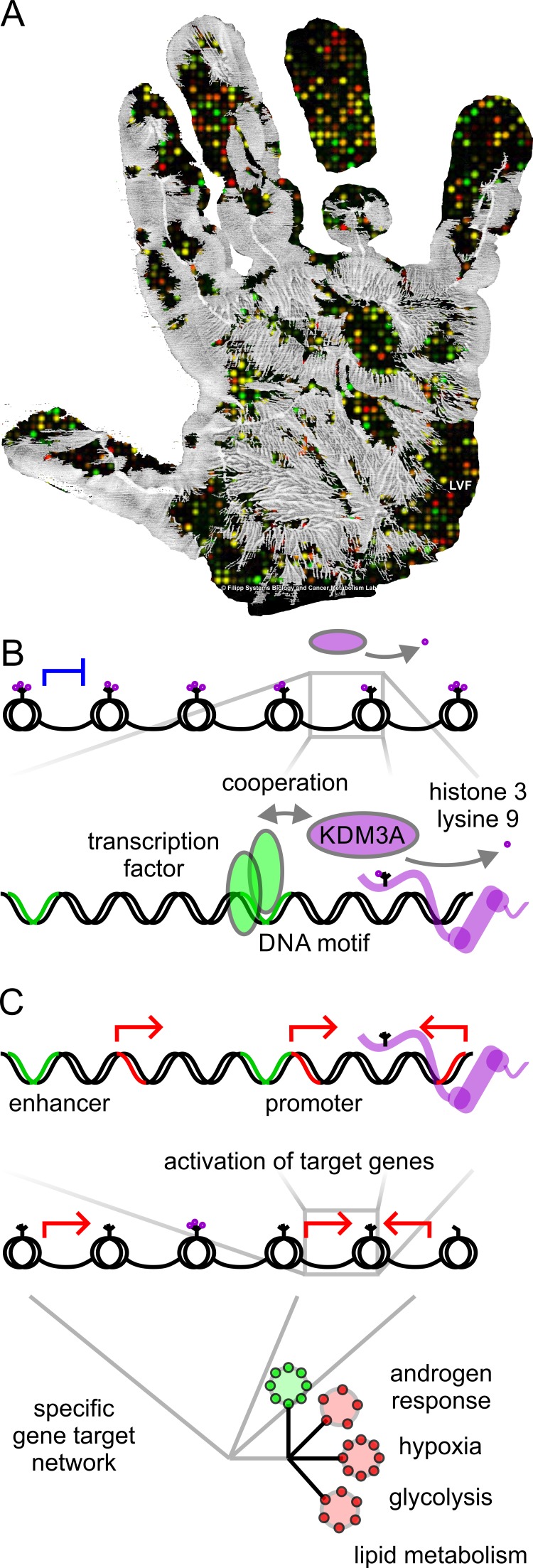
Network activation and oncogenic role by the epigenomic master regulator *KDM3A* **A.** Epigenomic handprint-Genome-wide impact of an epigenomic master regulator on a transcriptional network. **B.** The histone H3 lysine K9 demethylase KDM3A (also called JMJDlA or JHDM2A) has the biochemical activity to remove repressive epigenetic marks. Cooperation between transcription factors and epigenetic remodelers allows for transcriptional modulation and target specificity. **C.** Activation of target genes by transcriptional cooperation enables pathway enrichment of signaling and metabolic gene target networks. Targeted DNA sites are shown as close-up representations of transcriptionally active euchromatin.

Genomics has changed the way we diagnose and treat cancer [1]. Our ability to map our own genes (Figure 1A) will be a bigger part of medical care in the future. But perception, value, and risks of personal genomics are ongoing topics of controversial discussion [2]. What if conclusions extracted from the genomic data are wrong? What if people make life-altering decisions just because of a test that delivers statistical probabilities?

However, there is more to a genome map than a linear DNA sequence and yet we might know less than we think. A recent study has published discoveries about an epigenetic factor named *KDM3A* (also called JMID1A or JHDM2A) [3]. The factor is a member of the large Jumonji family of histone demethylases, which has been assigned mysterious roles in cancer and cellular development. The study identified that epigenomic changes are not just a passive by-product of cancer. The epigenetic factor affects how an entire network of cancer genes behaves, and thereby taking on an oncogenic role. Further, the epigenetic factor cooperates and teams up with transcription factors to control specific gene target networks (Figure 1B-1C). Genome-wide binding studies using chromatin immunoprecipitation with next generation sequencing (ChiP-Seq) enabled global detection of epigenetic modifications and characterization of the epigenetic factor's footprint. Here, due to the ability to team up with transcription factors, the epigenetic factor concerts mitogenic and metabolic gene networks claiming the role of a cancer master regulator or epioncogene [4].

The Jumonji family of histone demethylases regulates transcription by removing methyllysine modifications from histone tails. Therefore, direct DNA interaction may not be required for the biochemical demethylase function. Amore efficient way to accomplish gene target specificity is by cooperating with transcription factors that recognize promoter and enhancer motifs via their DNA-binding domains (Figure 1B). Since tissue­ specific expression of transcription factors organizes and directs recognition of appropriate gene targets needed for cellular functions and development, cooperation with epigenetic modifiers provides an additional regulatory layer. The cooperating team of transcription factors and epigenetic modifiers has the ability to employ network­ specific epigenetic pattern. Thereby, loss of repressive epigenetic marks by KDM3A leads to transcriptionally active euchromatin and tumor-promoting gene activation.

A predominant signature of androgen-dependent signaling plays a key role in prostate cancer progression and its resistance to androgen-deprivation therapy, which is the primary treatment for metastatic or locally advanced disease [5]. Transcriptome and epigenome-wide assays could provide molecular insight of which regulators are active to drive gene expression and cancer progression. In the present study, KDM3A is found to activate a transcriptional network in androgen response, hypoxia, glycolysis, and lipid metabolism (Figure 1C) [3]. The preclinical research leads to potential new direction for epigenetic biomarker and cancer drug discovery. In future, small molecules targeting oncogenic epigenomic master regulators or metabolic effector enzymes can complement ongoing androgen-focused efforts to battle prostate cancer.

Many of the factors controlled by the epigenomic regulator KDM3A are well-known biomarkers such as the prostate-specific antigen (PSA). Why can we not create a simple test that tells us if we have good genes but an unfavorable epigenome? Our epigenome is highly dynamic. Epigenomic regulators, including Jumonji family members of histone demethylases, remove or add chemical marks allowing for transient gene read-outs while blocking it in the next minute [4]. Personal gene tests for prostate cancer exist. Abnormally high levels of PSA in the blood can mean that a man has prostate cancer. However, PSA testing cannot distinguish between low-risk and high-risk prostate cancer, and thus leads to overdiagnosis and overtreatment. In addition, such biomarker tests do not take epigenetic factors into account [6].

An additional complication is that gene activity can respond to the environment. Epigenetic silencing can, for example, arise as diet-induced consequence of inflammation [7]. Typical drug receptors are simply switched off in an attempt to heal the tissue, despite the underlying tumor may benefit from such hiding mechanisms. Without doubt, once we have a better understanding of epigenomic regulation, we will be able to design drugs that counteract these factors.
